# Maternal Obesity and Obstetric Outcomes in a Socially Vulnerable Region Within Brazil’s Unified Health System

**DOI:** 10.7759/cureus.99278

**Published:** 2025-12-15

**Authors:** Juliana A Dias, Isabela C Guimarães, Henrique César L Neves, Kinulpe Honorato-Sampaio

**Affiliations:** 1 Faculdade de Medicina and Programa de Pós-Graduação em Ciências da Saúde, Universidade Federal dos Vales do Jequitinhonha e Mucuri, Diamantina, BRA; 2 Programa de Pós-Graduação em Ciências da Saúde, Universidade Federal dos Vales do Jequitinhonha e Mucuri, Diamantina, BRA; 3 Faculdade de Medicina, Universidade Federal dos Vales do Jequitinhonha e Mucuri, Diamantina, BRA

**Keywords:** childbirth, gestation, health center, high-risk pregnancy, obesity

## Abstract

Introduction

Obesity is a major global public health challenge and a growing concern among women of reproductive age. In Brazil, more than half of these women are overweight, and obesity substantially increases the risk of gestational complications, particularly hypertension (HTN), diabetes, and cesarean delivery. High-risk prenatal care centers within the Brazilian Unified Health System (Sistema Único de Saúde, SUS) play a critical role in managing these conditions, especially in socioeconomically vulnerable regions. This study assessed the association between maternal obesity and childbirth outcomes in a high-risk prenatal care center serving the Jequitinhonha Valley, one of the country’s most socially vulnerable areas.

Methods

We conducted an institutional, cross-sectional study based on medical records from a State Specialized Care Center and its referral maternity hospital in Diamantina, Minas Gerais, Brazil. Pregnant women receiving high-risk prenatal care between 2021 and 2022 were included if they delivered at the referral hospital. Women with miscarriage, multiple gestation, or delivery elsewhere were excluded. Maternal, obstetric, and neonatal variables were analyzed across BMI categories according to WHO criteria. Statistical analyses included chi-square or Fisher’s exact tests, Kruskal-Wallis tests, and multiple linear regression. A significance level of p < 0.05 was adopted.

Results

Of 492 women assessed, 285 met the inclusion criteria. Obesity was prevalent in 56.5% of the sample. Obese women had significantly higher rates of cesarean delivery (64%) compared to overweight and eutrophic women (p = 0.046), with obese women being 1.6 times more likely to undergo a cesarean than overweight women and 2.6 times more likely than eutrophic women. Although the need for labor induction did not differ by BMI, indications for induction varied significantly, with hypertensive disorders occurring almost exclusively among overweight and obese women (p = 0.003). Neonatal outcomes, including Apgar scores and birth weight, did not differ significantly between BMI groups. Regression analysis showed gestational age (β = 0.427) and maternal BMI (β = 0.205) as independent predictors of birth weight. NICU admission was modest (1.75%), occurring mainly among premature infants of obese mothers.

Conclusion

Maternal obesity was associated with increased obstetric intervention, particularly cesarean delivery and induction for hypertensive disorders. Despite this, neonatal outcomes were comparable across BMI categories, suggesting a potential protective effect of specialized high-risk prenatal care. These findings highlight the importance of strengthening structured, multidisciplinary obstetric services within the SUS, especially in vulnerable regions. Expanding access to reproductive planning and targeted prenatal care may help reduce adverse maternal outcomes. Future longitudinal studies are needed to identify which components of specialized care most effectively mitigate the risks associated with gestational obesity.

## Introduction

Obesity is a chronic condition characterized by excessive body fat accumulation and associated with adverse physical and mental health outcomes [1]. It is a complex condition to manage and is recognized as one of the leading global public health challenges, responsible for a substantial burden of morbidity and mortality and closely linked to chronic diseases such as diabetes, metabolic syndrome, cancer, and cardiovascular disorders [2]. Its etiology is multifactorial, marked by significant heterogeneity across phenotypes influenced by genetic, molecular, environmental, social, and economic factors [3].

In Brazil, an estimated 55.2% of women of reproductive age are overweight, including 33.3% classified as overweight and 21.9% as obese [4]. Despite this high prevalence, obesity among women of reproductive age remains frequently neglected, in part due to the scarcity of specific, evidence-based therapeutic approaches [5]. The global upward trend in overweight and obesity during pregnancy poses a mounting challenge for health systems, requiring structured and comprehensive responses [6]. Pregnant women with overweight or obesity are at increased risk of complications such as gestational hypertension, gestational diabetes mellitus, and cesarean delivery [7,8].

Overweight and obesity can be diagnosed using the BMI, which is calculated by dividing weight (kg) by height squared (m²). Although recent studies have highlighted its limitations, BMI remains a recommended measure by the WHO for epidemiological and population monitoring purposes [9]. According to WHO definitions, individuals with a BMI of 25 or higher are classified as overweight and those with a BMI of 30 or higher as obese, cutoff points that remain standard for public health surveillance [10]. The repercussions of obesity during pregnancy are considerable: each 5-7-unit increase in BMI significantly elevates the risk of high-risk obstetric conditions [11].

High-risk pregnancy is characterized by maternal, fetal, or placental factors that compromise or may compromise the well-being of the mother and fetus, presenting conditions that potentially threaten maternal and/or fetal health and require specialized care. These conditions include unfavorable demographic characteristics (age <15 or >35-40 years, obesity, malnutrition), adverse reproductive history (recurrent miscarriage, perinatal death, previous preterm birth, prior gestational diabetes), severe chronic maternal diseases (heart disease, hypertension, pre-gestational diabetes, nephropathies, pneumopathies, infections such as HIV/tuberculosis), and current gestational complications (preeclampsia, gestational diabetes, fetal growth restriction, placenta previa, cervical insufficiency, fetal malformations, multiple gestation, and Rh isoimmunization) [12].

In this context, specialized high-risk prenatal care centers play a strategic role within the Brazilian Unified Health System (Sistema Único de Saúde, SUS). They are essential for the surveillance and proper management of pregnancies involving clinical conditions or risk factors that may compromise maternal and fetal health. These services are particularly relevant in regions marked by socioeconomic vulnerability and unequal access to healthcare. It is estimated that 10% to 20% of pregnancies worldwide are considered high risk, requiring intensive, continuous follow-up by trained multidisciplinary teams [13].

Evidence from a socially vulnerable Brazilian municipality shows that the presence of specialized obstetric care services is crucial for reducing maternal and neonatal complications, particularly when integrated with public health policies such as the Prenatal and Birth Humanization Program and the Stork Network Program [14]. By strengthening specialized high-risk prenatal care centers, these policies help mitigate regional inequities, expand access to qualified care, and promote longitudinal monitoring of pregnant women. This is particularly important in regions such as the Jequitinhonha Valley, which faces high poverty levels and significant challenges in accessing healthcare services [15].

In this context, the present study aimed to evaluate the association between obesity and childbirth outcomes among pregnant women receiving care at a specialized high-risk prenatal center located in the Jequitinhonha health macro-region, in the municipality of Diamantina, Minas Gerais, Brazil. The relevance of this study lies not only in the scarcity of scientific evidence focused on the realities of rural or inland regions of developing countries but also in its potential to offer concrete insights for improving obstetric care within the SUS. In vulnerable territories, the qualified performance of specialized centers may represent the difference between risk and protection for maternal and infant health.

## Materials and methods

The present study was an institutional, cross-sectional study based on medical records from a State Specialized Care Center (Centro Estadual de Atenção Especializada, CEAE) and its referral maternity hospital, both located in the municipality of Diamantina, Minas Gerais, Brazil. Reporting followed the Strengthening the Reporting of Observational Studies in Epidemiology (STROBE) guidelines [16]. The study protocol was approved by the Research Ethics Committee of the Federal University of the Jequitinhonha and Mucuri Valleys (approval number 3.564.077).

Diamantina serves as a regional healthcare hub in the Jequitinhonha Valley, a region historically marked by socioeconomic vulnerability and significant barriers to accessing healthcare. The local CEAE serves as a referral center for the outpatient management of high-risk pregnancies and aims to expand access to specialized services while strengthening priority care pathways within the Brazilian Unified Health System, particularly maternal and child health. Its mission is to reduce morbidity and mortality from preventable conditions associated with noncommunicable diseases. The service offers multidisciplinary care and specialized examinations, structured to ensure continuity of care and clinical resolution.

The referral obstetric unit for childbirth among women followed at the CEAE is Hospital Nossa Senhora da Saúde, a philanthropic institution contracted by the SUS. The hospital is part of Rede Cegonha, Brazil’s national maternal and child healthcare network, which promotes safe, evidence-based perinatal care. It is also accredited as a Baby-Friendly Hospital under the international Baby-Friendly Hospital Initiative. The hospital has 26 obstetric beds and serves high-risk pregnant women from 34 municipalities across the Jequitinhonha Valley. It also functions as a teaching site for health sciences programs at a public university.

Data collection was conducted through the review of medical records of pregnant women receiving high-risk prenatal care at the CEAE between 2021 and 2022. Subsequently, electronic medical records from the same patients were accessed at the referral maternity hospital to identify obstetric outcomes. The sampling frame consisted of all pregnant women referred to the high-risk prenatal center and its referral maternity hospital during the study period. No sampling was applied; all eligible medical records were included. Data extraction was conducted manually using a standardized form developed for this study. All data were organized into spreadsheets, and participant confidentiality and anonymity were ensured by removing identifying information immediately after data extraction. As this study involved the analysis of de-identified secondary data, informed consent was not required.

Women were included if they received prenatal care at the CEAE and delivered at Hospital Nossa Senhora da Saúde during the study period. Exclusion criteria were miscarriage, multiple gestation, or delivery occurring at another institution. Miscarriages and multiple gestations were excluded because they do not progress to the obstetric outcomes of interest and follow distinct clinical trajectories that could introduce undue heterogeneity into the analysis.

Both outpatient and hospital variables were assessed. Outpatient variables included maternal age, nutritional status (weight and BMI in the first and third trimesters), comorbidities, pregnancy planning, and first-trimester ultrasonography. Hospital variables included gestational age at delivery, need for labor induction, indication for induction (gestational diabetes mellitus (GDM); hypertension (HTN)/preeclampsia; other: premature rupture of membranes, thrombocytopenia, post-term pregnancy, oligohydramnios, hydronephrosis, fetal growth restriction, and macrosomia), mode of delivery (vaginal or cesarean), medications used for induction (misoprostol and/or oxytocin), duration and dose of misoprostol, induction failure, Apgar scores at one and five minutes, newborn birth weight, and NICU admission. Numerical variables were expressed as means and standard deviations, whereas categorical variables were presented as absolute and relative frequencies. Missing data were handled by complete-case analysis; records with unavailable information for a given variable were excluded from that specific analysis.

Hypertensive disorders and GDM were diagnosed according to the Brazilian Ministry of Health guidelines routinely applied in primary care and confirmed at the high-risk prenatal center. GDM diagnosis followed the 75-g oral glucose tolerance test (OGTT) thresholds (fasting ≥92 mg/dL, 1 h ≥180 mg/dL, 2 h ≥153 mg/dL). Other obstetric conditions, such as premature rupture of membranes, oligohydramnios, fetal growth restriction, and macrosomia, were classified according to standardized clinical criteria used by the maternity hospital.

For comparative analyses, women were categorized into three nutritional status groups according to WHO BMI criteria: eutrophic (BMI < 25), overweight (BMI ≥ 25 and < 30), and obese (BMI ≥ 30). Maternal nutritional status was assessed using first-trimester BMI, calculated from weight and height measured during the initial prenatal visit at the high-risk specialty center. These measurements were obtained directly by trained healthcare professionals.

The sample size was determined a priori using G*Power version 3.1.9.7 (Universität Düsseldorf, Germany). A chi-square goodness-of-fit test for contingency tables was used as the statistical model. A medium effect size (w = 0.30) was selected based on Cohen’s conventions, representing differences of practical relevance in obstetric outcomes while avoiding the unrealistically large sample sizes required to detect small effects. Degrees of freedom (df = 4) corresponded to the contingency table structure used in the main analyses (three BMI categories × two outcome categories). Using α = 0.05 and power = 0.80, the minimum required sample size was 133 participants.

To investigate whether nutritional status (eutrophic, overweight, or obese) was associated with categorical variables, the chi-square (χ²) test or Fisher’s exact test (when expected frequencies were < 5) was applied. Continuous variables were evaluated using the Shapiro-Wilk test for normality. Because the data did not follow a parametric distribution, comparisons between means were performed using the Kruskal-Wallis test. Multiple linear regression analyses (enter method) were conducted to examine the extent to which maternal age, gestational age, and BMI were associated with Apgar scores (1 and 5 minutes) and newborn birth weight. Model assumptions were assessed by examining residual distributions and collinearity diagnostics (variance inflation factors). The analyses were exploratory and aimed at estimating associations rather than predicting outcomes. Some potentially relevant confounders, such as interpregnancy interval, prepregnancy BMI, gestational weight gain, history of prior cesarean section, and substance use (alcohol or illicit drugs), were considered during study planning but were not consistently available in the medical records and therefore could not be included in the final multivariable models.

Statistical analyses were conducted using the open-source jamovi software (version 2.3.28) or JASP (0.95.4.0). The significance level was set at p < 0.05. The dataset used in this study is publicly available in the Figshare repository.

## Results

Between 2021 and 2022, 492 pregnant women received high-risk prenatal care at the CEAE. Of these, 207 were excluded due to miscarriage (n = 3), multiple gestation (n = 27), or delivery occurring in another municipality (n = 177). Thus, a total of 285 women comprised the final study sample.

Table 1 presents maternal characteristics, including age, chronic HTN and diabetes mellitus, pregnancy planning, first-trimester ultrasonography, and indications for labor induction. Nearly half of the women were 29 years old or younger. Among the comorbidities, 13.3% had chronic HTN and 4.4% had pregestational diabetes mellitus. Most pregnancies (58.8%) were unplanned. First-trimester USG, recommended for accurate dating of gestational age, was performed in 87.2% of cases. Approximately 45% of the women required labor induction.

**Table 1:** Profile of patients treated at the CEAE high-risk prenatal care unit who delivered at Hospital Nossa Senhora da Caridade, Diamantina, Jequitinhonha Valley, Minas Gerais, Brazil, between 2021 and 2022. CEAE: Centro Estadual de Atenção Especializada.

Characteristics	N (%)
Age	
<18	4 (2.0)
18-29	96 (47.3)
30-39	89 (43.8)
40-49	14 (6.9)
Chronic hypertension	
Yes	27 (13.3)
No	176 (86.7)
Pregestational diabetes	
Yes	9 (4.4)
No	194 (95.6)
Planned pregnancy	
Yes	75 (41.2)
No	107 (58.8)
First-trimester ultrasound	
Yes	177 (87.2)
No	26 (12.8)
Labor induction	
Yes	131 (45.0)
No	160 (55.0)

Table 2 shows the comparison of maternal characteristics and obstetric outcomes according to BMI classification. Women with obesity were predominant, representing 56.5% (n = 161) of the sample. Overweight women accounted for 31.5% (n = 90), and eutrophic women for 12% (n = 34). No differences were identified regarding gestational age at delivery or maternal age.

**Table 2:** Maternal and neonatal outcomes according to BMI classification from patients treated at the CEAE high-risk prenatal care unit who delivered their babies at Hospital Nossa Senhora da Caridade, Diamantina, Jequitinhonha Valley, Minas Gerais, Brazil, from 2021 to 2022. Numerical variables were expressed as means ± SDs, and categorical variables as absolute frequency (relative frequency), and standardized residuals; *P-values derived from chi-square/Fisher’s exact test or Kruskal-Wallis test; #premature rupture of membranes, thrombocytopenia, post-term pregnancy, oligohydramnios, hydronephrosis, fetal growth restriction, and macrosomia. CEAE: Centro Estadual de Atenção Especializada.

Outcomes	Variables	Eutrophic (n = 34)	Overweight (n = 90)	Obese (n = 161)	Statistics	P-value*
Maternal	Maternal age (years)	28.4 ± 5.5	29.4 ± 6.6	30.0 ± 6.3	1.685	0.431
	Gestational age (weeks)	38.3 ± 1.6	38.8 ± 1.5	38.5 ± 1.8	3.488	0.175
	Mode of delivery					
	Vaginal	22 (13.6), -1.80	56 (34.6), -1.24	84 (51.8), 2.30	6.176	0.046
	Cesarean	7 (6.6), 1.80	29 (27.4), 1.24	70 (64.0), -2.30		
	Labor induction					
	Yes	13 (10.2), -0.29	37 (29.1), -0.86	77 (60.6), 1.00	1.005	0.65
	No	16 (11.4), 0.29	48 (34.0), 0.86	77 (54.6), -1.00		
	Induction indication					
	Hypertension/preeclampsia	0 (0.0), -2.24	5 (14.7), -2.26	29 (85.3), 3.47	15.44	0.003
	Gestational diabetes mellitus	6 (10.0), -0.40	23 (38.3), 2.22	31 (51.6), -1.84		
	Other#	6 (20.0), 2.20	9 (30.0), 0.02	15 (50.0), -1.35		
	Induction failure					
	Yes	3 (9.4), -0.20	9 (28.1), -0.18	20 (62.5), 0.29	0.093	1
	No	10 (10.6), 0.20	28 (29.8), 0.18	56 (59.6), -0.29		
	Oxytocin use					
	Yes	11 (11.0), 0.55	30 (30.0), 0.41	59 (59.0), -0.72	0.595	0.743
	No	2 (7.4), -0.55	7 (25.9), -0.41	18 (66.7), 0.72		
	Misoprostol use					
	Yes	7 (8.6), -0.73	22 (26.8), -1.03	53 (64.6), 1.41	2.015	0.365
	No	6 (12.5), 0.73	17 (35.4), 1.03	25 (52.1), -1.41		
	Misoprostol dose (mcg)	42.9 ± 12.2	62.0 ± 42.6	69.9 ± 43.8	2.364	0.307
	Misoprostol duration (h)	14.1 ± 7.47	20.5 ± 13.47	20.1 ± 11.59	1.648	0.439
Neonatal	Apgar 1 min					
	<7	0 (0.0), -1.37	7 (43.8), 1.00	9 (56.2), -0.13	2.37	0.339
	≥7	22 (10.5), 1.37	66 (31.6), -1.00	121 (57.9), 0.13		
	Mean	8.2 ± 1.3	8.0 ± 1.4	8.23 ± 1.3	1.777	0.411
	Apgar 5 min					
	<7	0 (0.0), -0.47	0 (0.0), -0.98	2 (100.0), 1.21	1.475	0.623
	≥7	22 (9.9), 0.47	73 (32.7), 0.98	128 (57.4), -1.21		
	Mean	9.2 ± 0.7	9.1 ± 0.6	9.05 ± 0.9	4.003	0.135
	Birth weight (g)	3035 ± 447	3095 ± 487	3182 ± 568	6.786	0.034

Mode of delivery differed significantly across BMI groups, with a higher proportion of cesarean sections among obese women (64%) (χ²(2) = 6.176, p = 0.046; Cramer’s V = 0.152). Odds ratio analyses indicated that obese women were 1.6 times more likely to undergo a cesarean section than overweight women, and 2.6 times more likely than eutrophic women. In contrast, no significant differences were found across nutritional status groups in terms of the need for labor induction or induction failure.

However, BMI classification was significantly associated with the distribution of induction indications (χ²(4) = 15.44, p = 0.003; Cramer’s V = 0.249). The most frequent comorbidities leading to induction were hypertensive disorders and diabetes. Other indications (including premature rupture of membranes, thrombocytopenia, post-term pregnancy, oligohydramnios, hydronephrosis, fetal growth restriction, and macrosomia) were grouped due to the small numbers of cases. Indications related to HTN and preeclampsia occurred exclusively among overweight and obese women. Odds ratio analysis showed that obese women had 2.9 times higher odds of having labor induction due to HTN or preeclampsia compared with overweight women.

Regarding induction medications, there were no significant differences between BMI groups in the use of oxytocin or misoprostol, nor in misoprostol dose or duration of use. Neonatal assessment indicated that most newborns presented Apgar scores ≥ 7 at one and five minutes, with mean scores of 8 and 9, respectively, across all nutritional status groups. Maternal age, gestational age, and BMI did not significantly influence Apgar scores at one minute (F(3,149) = 0.432, p = 0.731; adjusted R² = 0.009; Root Mean Square Error (RMSE) = 1.220) or at five minutes (F(3,149) = 0.624, p = 0.601; adjusted R² = 0.012; RMSE = 0.820).

Regarding newborn weight, the Kruskal-Wallis test showed significant differences across BMI groups, with higher birth weights observed in the obesity group. When assessing predictors of birth weight, multiple regression analyses indicated that both gestational age and maternal BMI significantly influenced newborn weight (F(3,159) = 12.36, p < 0.01; adjusted R² = 0.231; RMSE = 418.6). Gestational age had the strongest effect (β = 0.427, p < 0.01), followed by BMI (β = 0.205, p = 0.004). Maternal age was not associated with newborn weight (β = 0.049, p = 0.488).

Regarding NICU admission, only five newborns (1.75%) required NICU care. Of these, four were born to obese mothers, were premature, and delivered via cesarean section. Due to the low number of NICU admissions, no further associations with other study variables could be performed.

## Discussion

In this study, our findings underscore the complexity of maternal obesity and its clinical implications for obstetric management. We observed a significant association between obesity and a higher occurrence of gestational complications, particularly hypertensive disorders, among women with elevated BMI, which frequently served as indications for labor induction. We also found a higher rate of cesarean deliveries among women with obesity, a result consistent with previous studies identifying excess body weight as a relevant obstetric risk factor [17]. No statistically significant differences were observed between nutritional status groups for neonatal Apgar scores. BMI influenced newborn weight; however, the effect size was small. This pattern may suggest that the specialized prenatal care provided by the high-risk service could contribute to more standardized identification and management of complications, helping to moderate some adverse outcomes traditionally associated with obesity. These findings highlight the importance of structured and qualified obstetric care within SUS, particularly in secondary and tertiary care settings, which are crucial for reducing maternal-infant health inequities.

The increasing prevalence of obesity among pregnant women represents an important public health challenge, especially within the context of obstetric care provided by the SUS. Existing literature indicates that women with obesity face higher risks of intrapartum complications, including labor dystocia, emergency cesarean sections, and postpartum hemorrhage [18,19]. Furthermore, these women frequently require labor induction with higher doses of cervical-ripening medications. The mechanisms underlying this phenomenon are not fully understood, but may involve alterations in uterine contractility associated with metabolic dysfunction [20,21].

The high prevalence of obesity in this study population mirrors national and international trends, reflecting broader social and economic changes that have influenced the nutritional profile of women of reproductive age [22]. Obesity is strongly associated with increased risk of adverse pregnancy events such as miscarriage, gestational diabetes, and preeclampsia, conditions that affect both maternal and fetal health and often lead to increased medicalization of childbirth, including higher rates of cesarean sections and unnecessary obstetric interventions [21]. These trends reinforce the urgency of intersectoral public policies aimed at promoting healthy eating and preventing obesity among women prior to conception.

Another concerning finding was the high rate of unplanned pregnancies, affecting nearly 60% of participants. This reflects persistent gaps in access to, and adherence to, effective contraceptive methods, particularly among women with obesity. As noted by Simmons KB and Edelman AB (2015) [23], reproductive planning is essential for reducing perinatal risks, especially in high-risk pregnancies. Strengthening family planning interventions in primary healthcare settings is therefore crucial, including individualized counseling, health education, and universal access to safe and effective contraceptive methods.

The high proportion of women who underwent first-trimester USG is a positive finding, as early gestational dating is essential for determining the optimal timing of induction. In our study, approximately half of all women required labor induction, often due to comorbidities such as HTN and diabetes, conditions strongly associated with obesity [24]. These findings reinforce obesity as a determining factor for obstetric interventions. Nevertheless, when appropriately indicated and conducted according to updated protocols, labor induction can reduce the need for cesarean sections and improve maternal outcomes, as recognized by the WHO [25].

We also observed a higher cesarean rate among women with obesity, consistent with prior evidence highlighting excess weight as an obstetric risk factor [17]. Yet, in our study, we did not identify statistically significant differences in Apgar scores among eutrophic, overweight, and obese groups. Other studies have reported increased risk of macrosomia and poorer neonatal outcomes in pregnancies complicated by obesity [26]. Although BMI influenced birth weight, the effect size was modest. Given the cross-sectional design, causal relationships cannot be inferred; however, the findings raise the possibility that systematic, multidisciplinary care within a high-risk referral system may help mitigate some adverse effects of gestational obesity, an interpretation that longitudinal studies should further examine. As highlighted by Giouleka S et al. (2023) [27], structured and patient-centered care is crucial in improving outcomes in this population.

Our results also showed that newborns requiring neonatal intensive care were predominantly born to obese mothers and were premature. However, the overall rate of NICU admission was very low (below 2%) compared with other studies [28,29], limiting statistical power for this outcome. These same studies identify maternal diabetes and HTN as significant risk factors for NICU admission.

We emphasize the importance of high-risk prenatal care centers in socioeconomically vulnerable regions such as the Jequitinhonha macro-region in Minas Gerais, which faces persistent barriers to healthcare access. The integrated work of obstetricians, nurses, nutritionists, and psychologists enables a more resolutive and humanized approach, essential for improving maternal and perinatal outcomes [30]. Strengthening the maternal and child healthcare network within the SUS, notably by expanding specialized services in underserved areas, is a priority strategy for reducing inequities and promoting health equity.

This study has limitations. A potential selection bias may be present, as only women who delivered at the referral maternity hospital were included, which may limit the representativeness of women followed at the CEAE who delivered elsewhere or did not progress to delivery. The hospital is a high-risk referral center and a Baby-Friendly Hospital, which may influence the clinical profile of the population served. BMI was measured during early pregnancy rather than before conception, which may introduce some degree of misclassification, although first-trimester measures are commonly used when prepregnancy data are unavailable. Residual confounding cannot be excluded, as several potentially important variables, such as parity, history of hypertensive disorders or diabetes, gestational weight gain, prepregnancy BMI, interpregnancy interval, and substance use, were not consistently recorded in the medical charts and therefore could not be included in the regression models. Some neonatal outcomes, such as NICU admission, were infrequent, limiting statistical power and the precision of estimates for these analyses. Finally, although data extraction followed a standardized structured form, the retrospective use of medical records may be subject to variability in documentation quality across providers.

## Conclusions

In conclusion, our findings highlight gestational obesity as a clinically relevant factor influencing the course of pregnancy and childbirth. Maternal overweight and obesity were associated with important obstetric outcomes in this high-risk prenatal population. We also observed patterns suggesting that structured follow-up within a specialized high-risk referral system may potentially support the timely identification and management of complications. Nonetheless, given the cross-sectional design, any apparent protective effect of specialized prenatal care should be interpreted with caution. Future longitudinal or interventional studies are needed to determine whether such care truly exerts a protective influence on maternal and neonatal outcomes. Still, health system managers and clinicians should prioritize preventive strategies, expand access to reproductive planning, and strengthen the quality of obstetric care across all levels of complexity.
